# Clinical Analysis of Analgesics and Steroids Use for Extraction of Teeth in Patients with Intellectual Disability Under General Anesthesia

**DOI:** 10.2174/1874210601711010181

**Published:** 2017-03-31

**Authors:** Shigeru Maeda, Yuka Honda, Hiroshi Tanimura, Yumiko Tomoyasu, Hitoshi Higuchi, Naomichi Murata, Masahiko Egusa, Takuya Miyawaki

**Affiliations:** 1 Department of Dental Anesthesiology, Okayama University Hospital, Okayama, Japan; 2 Department of Dental Anesthesiology and Special Care Dentistry, Okayama University, Graduate School of Medicine, Dentistry and Pharmaceutical Sciences, Okayama, Japan.; 3 Special Needs Dentistry, Okayama University Hospital, Okayama, Japan

**Keywords:** Acetaminophen, Betamethasone, Extraction, Flurbiprofen, General anesthesia, Intellectual disability

## Abstract

**Background:**

The extraction of lower wisdom teeth is often performed under general anesthesia in patients with intellectual disabilities. However, the choice of analgesics has not yet been investigated.

**Objective:**

To analyze the use of analgesics during general anesthesia for extraction including lower wisdom teeth in patients with intellectual disabilities.

**Methods:**

This research is a retrospective observational study. The study population was composed of all patients presenting for extraction of lower wisdom teeth under ambulatory general anesthesia in the clinic of Special Needs Dentistry in Okayama University Hospital from April 2011 to March 2016. The distribution of the combination of analgesics and the relationship between the use of analgesics and the type of extraction were investigated.

**Results:**

One hundred and twelve cases were enrolled in this study. Intravenous injections of flurbiprofen, acetaminophen and betamethasone were used in 96 (85.7%), 12 (10.7%) and 26 cases (23.2%), respectively. Flurbiprofen is a non-steroid anti-inflammatory drugs (NSAIDs). Acetaminophen is an old analgesic, but an injection of acetaminophen is new, which was released in 2013 in Japan. And betamethasone is not an analgesic, but a steroid. Betamethasone was used in combination with other analgesics, and was used at a higher dose in a case in which four wisdom teeth were extracted.

**Conclusion:**

Flurbiprofen was the main analgesic used for extraction of wisdom teeth under general anesthesia in patients with intellectual disabilities. Betamethasone was used to support flurbiprofen or acetaminophen for extractions of multiple wisdom teeth, with the aim of controlling swelling rather than relieving pain.

## INTRODUCTION

In dental treatment for patients with intellectual disabilities, pain management after treatment is a significant problem because of the patients’ inability to inform dentists or Care givers when pain is present [1]. Pain management after extraction of impacted teeth is especially important, since the pain may be quite severe [2].

Extraction of impacted teeth is often performed under general anesthesia in patients with intellectual disabilities [3], and requires different pain management than extraction under local anesthesia. During general anesthesia, patients are totally unconscious, and they do not feel any pain. Preemptive analgesia is essential to control the pain when patients emerge from general anesthesia. Additionally, because a venous line is inserted for general anesthesia, the analgesics can be administered intravenously instead of orally. Alternatively, a suppository can be administered while the patient is under general anesthesia. Thus, effective preemptive analgesia for the extraction of teeth under general anesthesia should be administered either by intravenous injection or by suppositories.

The analgesics currently available for intravenous administration are opioids, non-steroidal anti-inflammatory drugs (NSAIDs) and acetaminophen, as well as steroids to control swelling after teeth extraction [4]. As a suppository, opioids and NSAIDs are available. Although it is accepted that these analgesics can be used as pain management for the extraction of impacted teeth under general anesthesia, their use has never been investigated. We therefore investigated the use of the intravenous analgesia in a retrospective study.

## MATERIALS AND METHODS

### Study Design / Sample

To address the research purpose, the investigators implemented a retrospective observational study. The study population was composed of all patients presenting for extraction of lower wisdom teeth under ambulatory general anesthesia in the clinic of Special Needs Dentistry in Okayama University Hospital from April 2011 to March 2016. To be included in the study sample, general anesthesia with tracheal intubation had to be maintained with remifentanil. Patients were excluded as study subjects if hospitalization was planned.

### Variables

The extraction of teeth was categorized as follows: type 1; one lower wisdom tooth, type 2; upper and lower wisdom teeth on one side, type 3; both lower wisdom teeth, type 4; upper and lower wisdom teeth on both sides. Outcome variables were intravenous injection (yes or no) of flurbiprofen, acetaminophen, and betamethasone.

### Data Collection Methods

Data were collected from the clinical records of eligible patients. Patient information was de-identified and stored appropriately. This study was approved by the Ethics Committee, Okayama University Graduate School of Medicine, Dentistry and Pharmaceutical Sciences.

### Anesthetic Procedure

General anesthesia was initiated with insertion of an intravenous line. In cases in which an intravenous line was difficult to place, midazolam was given orally or sevoflurane was inhaled as induction, and occasionally both were used, followed by insertion of an intravenous line. Remifentanil started at 0.25 µg/kg/min, and propofol was continuously infused as a target-controlled infusion (TCI) with the target concentration initially set at 4.0 µg/ml. In patients with multiple drug allergies, sevoflurane was used instead of propofol. After loss of consciousness, Rocuronium was injected to obtain muscle relaxation, and an endotracheal tube was inserted through the nasal cavity.

Patients were continuously monitored with electrocardiography, blood pressure, SpO_2_, bispectral index (BIS) monitoring, and partial pressure of CO_2_ in an anesthetic circuit. Body temperature was measured every 30 min. After intubation, the infusion rate of remifentanil was reduced to 0.10 - 0.20 µg/kg/min. During treatment, the BIS value was maintained between 40 and 50 by adjusting the target concentration of propofol, or the concentration of sevoflurane, as described in a previous report [5]. Systemic blood pressure was maintained at no less than 80 mmHg. Prior to extraction of the teeth, local anesthetic containing 2% lidocaine and 1:80,000 adrenaline was used. During treatment, analgesics and/or steroids were used. After treatment, anesthetic infusion was terminated and the effect of the muscle relaxant was reversed with sugammadex if necessary. The endotracheal tube was removed when spontaneous breathing and consciousness was regained. We judged the patients’ recovery state using a post-anesthetic discharge scoring system [6] to estimate activity, vital signs, water intake, pain, and bleeding. Patients were permitted to be discharged when these factors had returned to the same level as on admission.

### Data Analysis

Pearson’s chi-square test was used to compare extraction type and outcome variables.

## RESULTS

One hundred and twelve cases were enrolled in this study (Table **1**), 81 males and 31 females. Median age was 25 years old (interquartile range or IQR: 15-49). Median body mass index (BMI) was 23.1 kg/m^2^ (IQR: 20.1-26.8). The median duration of the operation was 101 min (IQR: 85-122).

**Fig. (1):**
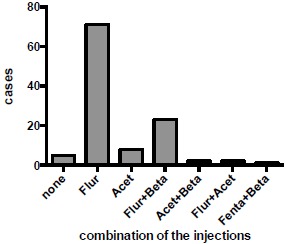
Distribution of usage of analgesics and steroids. Flur; flurbiprofen, Acet; acetaminophen, Beta; betamethasone, Fenta; fentanyl.

The intravenous analgesic agents used are shown in (Fig. **1**). Flurbiprofen alone was used in 71 cases (63.4%), and acetaminophen alone was used in 8 cases. A combination of flurbiprofen and betamethasone was used in 23 cases (20.5%). Two cases were administered with a combination of acetaminophen and betamethasone, and a further two were treated with flurbiprofen and acetaminophen. A combination of fentanyl and betamethasone was used in one patient who was allergic to multiple drugs. In 26 cases, betamethasone was used, always in combination with other analgesics. In five cases, no intravenous analgesia was used. No suppositories were used for any participants of this study.

Betamethasone was used in 80% of type 4 cases, involving extraction of upper and lower wisdom teeth on both sides, but in less than 25% of type 1, 2 and 3 cases. There was a significant difference in the usage of betamethasone between different types of extraction (Table **2**).

## DISCUSSION

In this study, flurbiprofen was used in 96 out of 112 cases, indicating that it plays a major role in pain management after extraction of wisdom teeth under general anesthesia in patients with intellectual disabilities in our facility. The effectiveness of flurbiprofen or NSAIDs in acute postoperative pain has been demonstrated by a previous systematic review paper [7, 8], and our results are consistent with this.

Of the 26 cases, in which betamethasone was used, 23 also used flurbiprofen, and 2 also used acetaminophen, suggesting that betamethasone was expected to support the effect of flurbiprofen and acetaminophen. Since corticosteroids have been demonstrated to be effective in relieving pain, swelling and trismus following surgical extraction of wisdom teeth [9, 10], the combination of flurbiprofen and betamethasone is thought to contribute to management of pain as well as swelling after extraction of teeth under general anesthesia. Five cases involved extraction of upper and lower wisdom teeth on both sides, and betamethasone was used in four of these. Since betamethasone exerts a strong anti-inflammatory effect after surgery, it is expected to control inflammation after more invasive treatment, such as extraction of four wisdom teeth. No side effects were reported with short-term use of corticosteroids in previous reports [10, 11]; suggesting that betamethasone could have been used in more cases of extraction of wisdom teeth, especially those involving bone removal.

Acetaminophen has been demonstrated to provide a statistically significant benefit when compared with placebo for pain relief and pain intensity without statistically significant difference in adverse events in meta-analysis. In addition, acetaminophen acts through TRPA1, a family of transient receptor potential ion channels, and inactivates voltage-gated calcium channel (VGCC) in the central terminals of primary sensory neurons in the dorsal horn [12], indicating that the mechanism of action of acetaminophen is totally different from that of NSAIDs. Therefore, a combination of acetaminophen and NSAIDs has been considered to confer additional analgesic efficacy compared with alone in an internal use [7]. Then, a combination of injection of NSAIDs and acetaminophen are considered to be also effective against teeth extraction under general anesthesia. In this study, acetaminophen was used only in 8 cases. However, it should have been used more cases since it is not easy for a part of patients in this study to inform suffering from pain and take medicines because of intellectual disabilities.

Moreover, addition of betamethasone to the injections of flurbiprofen and acetaminophen may provide ideal pain and swelling management in the extraction of impacted wisdom teeth under general anesthesia. As described above, action mechanisms of flurbiprofen and acetaminophen is different from each other, and betamethasone reduces other complications, such as swelling and trismus with other mechanisms of action. Besides, administration of analgesics during general anesthesia is intended to provide preemptive analgesia to effectively control pain after extraction of impacted teeth [13, 14]. Especially, acetaminophen injection is also known to be effective in controlling postoperative pain when used for preemptive analgesia [15, 16]. Thus, administration of injections of flurbiprofen, acetaminophen and betamethasone bring strong management for pain and other symptoms with minimum side effects for extraction of impacted third molars under general anesthesia.

Fentanyl was used only in one patient, who has allergy for multiple medicines. Opioid seems not to be preferably used for pain management after the extractions because of side effect, such as nausea and headache. In five cases in our study, no analgesic was administered as pain management, with no specific reason documented. Since it is not easy for a part of patients with intellectual disability to inform family members and/or caregivers of their pain and to take medicines of pain management, preemptive analgesia is strongly recommended in all these cases during treatment under general anesthesia. However, injections of all flurbiprofen, acetaminophen and betamethasone are not necessary for all cases of teeth extraction. Then, suitable combination of injections to each case is expected to shown in future clinical studies.

## CONCLUSION

Flurbiprofen was the main analgesic used for the extraction of wisdom teeth under general anesthesia in patients with intellectual disabilities. Betamethasone was used to support flurbiprofen or acetaminophen, with the aim of controlling swelling rather than relieving pain.

## Figures and Tables

**Table 1:** Patient background and summary of anesthetic data.

Category	Variables	Participants (N=112)
Study variables	Gender (male) n (%)	81 (72.3%)
	Age (y.o., IQR)	25 (20-32%)
	Body mass index (kg/m^2^, IQR)	23.1 (20.1-26.8%)
	Treatment time (min, IQR)	101 (85-122%)
	Categorization of extraction n (%)	
	Group 1 (one lower)	55 (49.1%)
	Group 2 (upper and lower of one side)	40 (35.7%)
	Group 3 (lower teeth of both sides)	12 (10.7%)
	Group 4 (4 teeth)	5 (4.5%)
Outcomes	Flurbiprofen n (%)	96 (85.7%)
	Acetaminophen n (%)	12 (10.7%)
	Betamethasone n (%)	26 (23.2%)
	Fentanyl n (%)	1 (0.9%)

Continuous variables were given as mean ± SD, and descriptive variables were given as %.

**Table 2:** Number of cases (%), in which the injections were used.

Extraction type	1	2	3	4	*P*-value
Total number of patients	55	40	12	5	
Flurbiprophen	46 (83.6%)	34 (85.0%)	11 (91.7%)	5 (100%)	0.708
Acetaminophen	8 (14.6%)	4 (10.0%)	0	0	0.407
Dexamethasone	9 (16.4%)	10 (25.0%)	3 (25.0%)	4 (80.0%)	0.014*
Fentanil	0	0	1 (2.5%)	0	0.038*

Chi-square test was used to compare groups.
